# Meetings of Societies

**Published:** 1894-06

**Authors:** 


### Bristol Medico-Chirurgical Society.

*January 10th*, 1894.

Mr. J. GREIG SMITH, President, in the Chair.

The following skin cases were exhibited: (1) by Dr. A. J. HARRISON, a man with non-specific psoriasis attacking unusual parts; (2) by Dr. BERTRAM ROGERS, a boy with extensive patchy psoriasis; (3) by Mr. W. DUNCAN, a woman with psoriasis which had been much improved by thyroid extract; (4) by Dr. C. ELLIOTT, a boy, aged 11, with complete alopecia.

The PRESIDENT showed specimens of ovarian cystic tumours with hemorrhages into walls of cysts and separation of pedunculated growths, etc.

Dr. J. E. SHAW gave the history of a girl, aged 19, who had suffered for two months from painful dyspepsia before her admission to the Infirmary, where she developed signs of general peritonitis. A diagnosis of perforating gastric ulcer was made, and Mr. PAUL BUSH performed a cœliotomy. The viscera appeared healthy, but a small quantity of purulent serum was found. The peritoneal cavity was irrigated. She did well for a time, but began to expectorate and showed signs of consolidation of left lower lobe of lung, and albumen appeared in the urine. After death, an ulcer was found in upper and anterior suface of the stomach, with a small perforation leading into a sub-diaphragmatic abscess-cavity which communicated with a ragged gangrenous cavity in the base of the left lung. The parotid and sub-maxillary glands were suppurating.

Dr. J. MICHELL CLARKE and Mr. C. A. MORTON described the case of a female child who suffered from pains in abdomen after food, and from constipation. She became collapsed, and a dull swelling was found over left iliac fossa. This was incised, and a cavity was found surrounded by matted-together intestines. There was a fæcal discharge into this cavity, which was irrigated and drained. The child recovered. This was considered to be a case of perforation of the colon by a small foreign body.

---

*February 14th*, 1894.

Mr. J. GREIG SMITH, President, in the Chair.

Mr. W. M. BARCLAY showed a girl with double congenital dislocation of the hip.

Dr. BERTRAM ROGERS showed a boy with general thinning of the hair following a patch of complete alopecia which had been quite cured. Chrysophanic acid seemed to increase the growth of the hair.

Dr. J. MICHELL CLARKE exhibited a specimen of perforated gastric ulcer on the border of a much larger ulcer which adhered to the pancreas.

Dr. F. H. EDGEWORTH described the case of a child, aged four years, who had suffered from recurrent attacks of jaundice, and for a year before death from an abdominal tumour in the region of the gall-bladder. Cholecystotomy was performed by Mr. Ewens, and twenty-nine ounces of greenish fluid removed. The patient died a week afterwards from cardiac failure, and at the *post mortem* it was found that the sac of the tumour was a greatly-distended common bile-duct. The liver was cirrhosed.

Dr. Bertram Rogers showed a round-celled sarcoma removed by Mr. Ewens from the broad ligament of a child two years old.

Dr. Theodore Fisher read a paper on adherent pericardium in children. (See page 93).

Mr. C. A. Morton exhibited a patient, aged 75, with a pulsating tumour on the sole of the foot, probably an aneurismal varix. Seven years ago the heel became swollen and black, and was incised. This was followed by ulceration, which got well, but recurred a year ago. The posterior tibial was ligatured, and a pulsating vein was felt in its neighbourhood. Remission of pain followed the operation. A loud bruit is now audible over the dorsalis pedis, and there is slight pulsation in the heel.

---

*March 14th*, 1894.

Mr. J. Greig Smith, President, in the Chair.

Mr. H. F. Mole exhibited three specimens of gastric ulcer. (1) A malignant ulcer of the pylorus, with a perforation posteriorly, causing an abscess in sac of lesser omentum. (2) Ulcer of pylorus with aneurism of pancreatico-duodenal artery, which had ruptured, causing death from hemorrhage. (3) Perforating funnel-shaped ulcer in anterior wall; also older ones posteriorly, adherent to pancreas.

The President showed—(1) The stomach-wound and the incision in the parietes, both perfectly healed, made in the second of the above cases. (2) The wound made in a case of gastrostomy, with firm adhesions between stomach and abdominal wall.

Dr. F. H. Edgeworth read notes on a case of syringomyelia. (See page 100.)

Dr. Walter Swayne described—(1) A case of labour which was obstructed by atresia of the cervical canal. This was dilated, and labour terminated normally. These cases were probably due to organisation of the clot formed in the canal during pregnancy. (2) The case of a child who died when two months of age from rupture of an enlarged spleen. The cause of the injury was probably over-lying by the mother.

Dr. J. Michell Clarke showed specimens from a case of lymphadenoma with deposits in the viscera and lymphatic glands. The patient was a girl aged 25, and the disease lasted three years. Microphotographs of sections were shown with the lantern.

Mr. W. M. Barclay described a case of intussusception in which cœliotomy was performed. The intussusception was easily reduced, but a longitudinal rent was found in the gut. This was sutured, but the patient sank. He also showed a concretion three-quarters of an inch long, composed of inspissated fæcal matter, bile pigments, etc., which had caused perforating appendicitis.

---

*April 11th*, 1894.

Mr. J. Greig Smith, President, in the Chair.

Dr. A. E. Aust Lawrence described a case of amenorrhœa in which there was marked pigmentation of the skin. (See page 107.)

Mr. C. A. Morton described a case of cervical fistula in a man aged 19. The fistula had been discharging and temporarily closing since the age of three. A cystic growth the size of a walnut, containing jelly-like fluid, was dissected out, and the patient recovered completely. These fistulæ originated in the canal leading from the foramen cæcum to the back of the hyoid bone.

The President showed a series of translucencies illustrating the growth of tumours. Thin sections, rendered semi-transparent by soaking in glycerine and boric acid, were mounted between plates of glass. Teratomata, adenomata, scirrhus of the breast, showing mode of extension of skin, glands, &c., various sarcomata, myomata, &c., were shown.

Mr. G. Munro Smith exhibited a series of lantern slides photographed by Dr. Imlay and Mr. J. Taylor from specimens of myoma of prostate, angioma of liver, epithelioma, scirrhus, &c.

---

*May 9th*, 1894.

Dr. E. Markham Skerritt in the Chair.

Dr. R. Shingleton Smith showed a specimen of splenic abscess from a girl who had suffered from vague abdominal symptoms and hectic temperature for more than a month before death, which resulted from wide-spread peritonitis. Cœliotomy was performed and the peritoneal cavity irrigated, but without any good results. There was evidence of old appendicitis and of pyo-phlebitis of the liver. The condition was probably pyæmic.

Mr. C. A. Morton described the case of an elderly woman who dislocated her spine between the sixth and seventh cervical vertebræ, with crushing of the cord extending as high as the fifth cervical. There was paralysis of lower limbs and intercostals and of the muscles of upper limbs, except supra- and infra-spinatus, deltoids, biceps, pronators, supinators, and subscapularis. The area of anæsthesia, as well as the paralysis, pointed to a lesion of the cord at the sixth nerve-roots, and this diagnosis was verified *post mortem*. He also showed a boy, aged 16, whose olecranon had been fractured by muscular action three weeks before he came to the Hospital. There was considerable movement. Probably it was a case of separation of epiphysis.

Dr. Arthur B. Prowse read notes on a case of fatal paludism in a young man who had never resided in a malarial district, but had travelled to the East Indies and West Coast of Africa. Quinine stopped the intermittent attacks, and the patient improved; but his temperature again rose to 103°, and remained high for a few days before his death, which was sudden. The *post mortem* threw no light on the fatal termination. Specimens of the spleen, &c., were shown, with the pigmented masses characteristic of the disease. Dr. Michell Clarke exhibited some of the malarial plasmodes in the blood of another patient.

Dr. P. Watson Williams exhibited some lantern slides of photographs by Dr. Imlay, of the Eberth-Gaffky bacillus from Agar-Agar cultures taken from ulcers in the larynx of a rapidly fatal case of typhoid fever. The cultures were made by Mr. F. W. Stoddart. It was thought that the patient contracted the disease from a man in the same ward with a typhoid ulcer of the larynx; it was probably transmitted by the breath and expectoration.

G. Munro Smith, *Hon. Sec.*

